# Interferon-Gamma Release Assay for the Diagnosis of Latent TB Infection – Analysis of Discordant Results, when Compared to the Tuberculin Skin Test

**DOI:** 10.1371/journal.pone.0002665

**Published:** 2008-07-16

**Authors:** Albert Nienhaus, Anja Schablon, Roland Diel

**Affiliations:** 1 Institution for Statutory Accident Insurance and Prevention in the Health and Welfare Services, Department of Occupational Health Research, Hamburg, Germany; 2 School of Public Health, Institute for Medical Sociology, Heinrich Heine University, Düsseldorf, Germany; Columbia University, United States of America

## Abstract

**Background:**

With the Interferon-γ release assays (IGRA) a new method for the diagnosis of latent tuberculosis infections (LTBI) is available. Due to the lack of a gold standard for the diagnosis of LTBI, the IGRA is compared to the Mantoux Tuberculin Skin Test (TST), which yields discordant results in varying numbers. Therefore we assessed to which extent discordant results can be explained by potential risk factors such as age, BCG vaccination and migration.

**Methods and Findings:**

In this pooled analysis, two German studies evaluating the Quantiferon-Gold In-Tube test (QFT) by comparison with the TST (RT23 of SSI) were combined and logistic regressions for potential risk factors for TST+/QFT− as well as THT−/QFT+ discordance were calculated. The analysis comprises 1,033 participants. Discordant results were observed in 15.4%, most of them being TST+/QFT− combinations. BCG vaccination or migration explained 85.1% of all TST+/QFT− discordance. Age explained 49.1% of all TST−/QFT+ discordance. Agreement between the two tests was 95.6% in German-born persons younger than 40 years and not BCG-vaccinated.

**Conclusions:**

After adjustment for potential risk factors for positive or negative TST results, agreement of QFT and TST is excellent with little potential that the TST is more likely to detect old infections than the QFT. In surveillance programs for LTBI in high-income, low TB incidence countries like Germany the QFT is especially suited for persons with BCG vaccination or migrants due to better specificity and in older persons due to its superior sensitivity.

## Introduction

The burden of tuberculosis (TB) in healthcare workers (HCW) remains high in low- and middle-income countries [1] as well as in high-income countries [1]–[5]. Therefore an efficient strategy for surveying exposed HCW or anyone else exposed to active TB patients is needed. With declining incidence of active TB, the purpose of these screening programs widened from early active TB detection to latent TB infection (LTBI) detection and preventive chemotherapy [6] below. For these screenings a specific test that allows for the diagnosis of recent TB infections [7] likely to progress into active TB is warranted.

For the diagnosis of latent tuberculosis infections (LTBI) the recently developed Interferon-γ release assays (IGRA) are good alternatives to the unspecific tuberculin skin test (TST) [8]–[11], which has been in use for nearly 100 years [12]. Due to the lack of a gold standard for the diagnosis of LTBI, IGRA is compared to TST in evaluation studies. In a meta-analysis of studies in healthy populations with varying risk for LTBI, discordant results between IGRA and TST were found in 21% (ELISpot, T-SPOT.TB) or 29% (ELISA, QuantiFERON-Gold) of the participants [13]. Among discordant results, TST-positive and IGRA-negative (TST+/IGRA−) combinations prevailed. This is easy to explain because the IGRA uses few *Mycobacteria tuberculosis*-specific antigens (ESAT-6 and CFP-10 of the region of difference, RD1) while the tuberculin of the TST is a mix of about 200 non-specific antigens that are shared with nontuberculous mycobacteria (NTM) as well as with the strains developed from *Mycobacterium bovis* used for bacilli Calmette-Guérin (BCG) vaccination [9], [11]. Nevertheless the higher rate of TST-positive results compared to those in IGRA might also indicate that the TST is more likely to detect resolved or old LTBI while the IGRA mainly detects current or recent infections [7], [14].

In a German contact-tracing study only those with a positive IGRA progressed towards active TB, while none of those with a positive TST but negative IGRA (TST+/IGRA−) developed TB in the two years following close contact to an active TB case [15]. As progression to active TB is higher in those with recent infections [16], this study supports the hypothesis that IGRA might rather detect new infections.

In the literature little attention is given to TST-negative but IGRA-positive results. Assuming the IGRA to be highly specific it is likely that this combination indicates LTBI [17]. A waning of the TST with age is discussed in literature [18]. Whether the IGRA is waning to the same extent with age as the TST is an open question.

So far the extent to which BCG vaccination and NTB explain TST+/IGRA− discordance is not analyzed and the reasons for positive IGRA that are not verified in the TST (TST−/IGRA+) are unknown [13], [19]. The proportion of TST+/IGRA− results that can not be explained by known risk factors might be explained by a higher sensitivity of the TST for old infections. In consequence the IGRA would be indicative of current or recent infections. If this rationale is true, a relevant proportion of TST+/IGRA− results that cannot be explained by BCG vaccination or exposure to NTM should be observed in a population of a country like Germany, which experienced the transition from high to low TB incidence in the last decades. In a country with a decreasing incidence of active TB, prevalence of LTBI in older age groups should be higher than in the younger, less exposed age groups. Again if the rationale is true, this effect should be more pronounced in the TST than in the IGRA. We analyzed risk factors for discordant results when the IGRA is compared to the TST in order to verify the hypothesis that IGRA is sensitive to recent or current infections while the TST is sensitive to both old and recent infections.

## Methods

For this analysis we combined two study populations consisting of 1,040 healthy persons. Due to indeterminate results in the IGRA 7 persons had to be excluded from the analysis. Out of the remaining 1,033 persons (table 1), 601 were part of the general population examined in the scope of contact tracing [20] and 432 were healthcare workers routinely screened for TB [21]. Both studies were carried out in the scope of German legislation concerning TB surveillance. They both used the same study protocol and were carried out by the same principal investigators (R.D., A.N.). Therefore they were suitable for a combined analysis. Information on BCG vaccination, country of birth, age, gender, and previous tests was collected in standardized interviews. BCG vaccination was verified by scars or vaccination records. In Germany, up until 1982 all newborns were BCG vaccinates. Thereafter vaccination was recommended only for newborns with high TB risk. No general recommendation on revaccination was issued [22]. Since 1998 BCG vaccination has no longer been recommended in Germany [23].

**Table 1:** Description of the study population.

	Mean	Standard deviation
Age*	N	%
1–29 years	490	47.4
30–39 years	226	21.9
40–49 years	246	23.8
50–68 years	71	6.9
Female	638	61.8
Foreign-born**	262	25.4
Previous BCG vaccination	448	43.4
Previous TST	312	30.2
HCW	432	41.8
TST >5 mm	311	30.1
TST ≥10 mm	191	18.5
TST ≥15 mm	69	6.7
QFT-positive	100	9.7
Total	1,033	100.0

* Age: mean 31.6, standard deviation 12.7.

** All but 6 emigrants were born in countries with a TB incidence (>20/100.000) well above that of Germany (<6/100.000), mainly Turkey, other Eastern European countries, or Africa.

In both study populations the TST was performed using 2-TU of PPD RT23 (Statens Serum Institute, Copenhagen, Denmark). The test was administered to the volar side of the forearm of the participants and read 72 to 96 hours after the application. The transverse diameter of the induration was measured. The observers were blinded to the IGRA results.

Before TST application, the standardized interview was performed and blood for the IGRA was drawn. For the IGRA, a variation of the QuantiFERON-TB Gold assay (Cellestis Limited, Carnegie, Australia), the QuantiFERON-TB Gold In-Tube test (QFT), was used. This whole-blood assay uses overlapping peptides corresponding to ESAT-6, CFP-10, and a portion of tuberculosis antigen TB7.7 (Rv2654). Stimulation of the antigenic mixture occurs within the tube used to collect the blood. Tubes were incubated at 37°C overnight before centrifugation, and INF-γ release was measured by ELISA following the protocol of the manufacturer. All the assays performed met the manufacturer's quality-control standards. The test was considered positive when INF-γ was ≥0.35 IU after correction for the negative control. Observers were blinded to the results of the TST.

Due to the lack of a gold standard, sensitivity and specificity were not calculated. The Pearson Chi-square test was used to compare frequencies of test results among different groups of participants. For ordered risks, the proportions of positive test results were compared using the chi-square test of trend. P<0.05 was considered to be statistically significant. Agreement and Kappa values were calculated for the two tests with varying cut-offs for a positive TST (>5 mm, > = 10 mm, > = 15 mm). Odds ratios (OR) for discordant test results depending on different putative predictive variables were calculated using logistic regression. Model building was performed backwards using the chance criteria for variable selection [24]. The expected number of discordant results was calculated by the product of the proportion of discordant results in the unexposed strata and the number of observations in the exposed strata. The difference between observed and expected discordance was considered as the proportion of the discordant results explained by the analyzed risk factor. The expected discordance was used to calculate a corrected agreement between TST and QFT.

Data analysis was performed using SPSS, Version 14 (SPSS Inc., Chicago, Illinois). The study protocols of both studies combined for this paper were approved by the ethics committee of the Hamburg Medical Council. All persons gave their written informed consent prior to their inclusion in the studies.

## Results

Table 1 describes the study population. 30.1% had an induration diameter in the TST of >5 mm and 18.5% had a diameter of 10 mm or more. The QFT was positive in 9.7%. For 5 participants born in Germany and not BCG-vaccinated, the induration diameter in the TST was ≥15 mm. All of these had a positive QFT (table 2). Kappa was influenced by BCG vaccination and birthplace. Kappa was lowest when a cut-off point >5 mm for TST was used and the participants were foreign-born and BCG-vaccinated (0.04). Kappa was highest in German-born, not BCG-vaccinated participants when ≥10 mm was used as cut-off point for the TST. Agreement between QFT and TST was best with a TST diameter of at least 15 mm as cut-off point (89.8%). But Kappa was best with at least 10 mm as cut-off point for the TST in the whole sample (0.37) and in the different subgroups (table 2). Therefore further analysis was carried out with 10 mm as cut-off point for the TST. Foreign birth was a risk factor for LTBI in both TST and QFT (table 3). The OR for BCG vaccination was 5.1 (95%CI 3.51–7.33) in the TST while the QFT was not affected by vaccination (OR 1.1; 95%CI 0.71–1.68). Prevalence of LTBI increased with age with the QFT (test for trend p<0.005), but not with the TST (p = 0.67).

**Table 2:** Positive results of the TST with different cut-off points confirmed by QFT for various subgroups and kappa-value.

	TST >5 mm/QFT+	TST ≥10 mm/QFT+	TST ≥15 mm/QFT+
BCG	n (TST)* [QFT]** kappa	n (TST)* [QFT]** kappa	n (TST)* [QFT]** kappa
No (n = 585)	38 (46.3) [69.1] 0.50	31 (60.8) [56.4] 0.54	11 (84.6) [20.0] 0.30
Yes (n = 448)	37 (16.2) [82.2] 0.12	35 (25.0) [77.8] 0.28	21 (37.5) [53.3] 0.34
Born			
Germany	40 (23.7) [70.2] 0.27	34 (36.6) [59.6] 0.40	14 (53.8) [24.6] 0.30
foreign born	35 (24.6) [81.4] 0.17	32 (32.7) [74.4] 0.29	18 (41.9) [41.9] 0.30
BCG vaccination and born			
no BCG, born in Germany	23 (50.0) [67.6] 0.54	18 (69.2) [52.9] 0.57	5 (100.0)[14.7] 0.24
no BCG, foreign born	15 (41.7) [71.4] 0.41	13 (52.0) [61.9] 0.48	6 (75.0) [28.6] 0.36
BCG, born in Germany	17 (13.8) [73.9] 0.13	16 (23.9) [69.6] 0.28	9 (42.9) [39.1] 0.37
BCG, foreign born	20 (18.9) [90.9] 0.04	19 (26.0) [86.4] 0.18	12 (34.3) [54.5] 0.27
All	75 (24.1) [75.0] 0.26	66 (34.6) [66.0] 0.37	32 (46.4) [32.0] 0.33

Agreement: TST >5 mm: 74.8% TST ≥10 mm: 84.2% TST ≥15 mm: 89.8%.

* (TST) = percentage of all TST+confirmed by QFT.

** [QFT] = percentage off all QFT+confirmed by TST.

**Table 3:** Adjusted Odds Ratios for TST and QFT depending on age, gender, migration, or BCG vaccination.

Age	TST > = 10 mm			QFT positive		
	N (row%)	OR	95%CI	N (row%)	OR	95%CI
1–29 years	91 (18.6)	1	–	31 (6.3)	1	–
30–39 years	39 (17.3)	1.3	0,82–2.03	18 (8.0)	1.4	0.77–2.62
40–49 years	47 (19.1)	1.4	0.93–2.22	37 (15.0)	2.9	1.78–5.01
50–68 years	14 (19.7)	1.6	0.82–3.28	14 (19.7)	4.2	2.06–8.59
p for trend	0.67			<0.0005		
Gender						
Female	104 (16.3)	1	–	57 (8.9)	1	–
Male	87 (22.0)	1.4	0.99–1.99	43 (10.9)	1.3	0.85–2.02
p-value	0.021			0.303		
Birthplace						
Germany	93 (12.1)	1	–	57 (7.4)	1	–
Other	98 (37.4)	4.6	3.21–6.53	43 (16.3)	2.6	1.71–4.09
p-value	<0.0005			<0.0005		
BCG						
No	51 (8.7)	1	–	55 (9.4)	1	–
Yes	140 (31.3)	5.1	3.51–7.33	45 (10.0)	1.1	0.71–1.68
p-value	<0.0005			0.729		

Using a diameter of 10 mm as cut-off point for the TST yielded 159 (15.4%) discordant test results (table 2); most of them had the combination TST+/QFT− (125 out of 159 or 78.6%). A previous BCG vaccination and being foreign born increased the probability of TST+/QFT− discordance (table 4) with statistically significant OR of 8.6 and 4.1, respectively. The highest proportion of TST+/QFT− was seen in foreign-born participants with a BCG vaccination (42.5% of all participants in these strata). The combination of a BCG vaccination and being foreign-born yielded an OR for TST+/QFT− discordance of 40.9 (95%CI 18.6–89.4) which was higher than the sum of the OR for birthplace (OR 5.4, 95%CI 2.2–13.5) and for BCG vaccination (OR 10.4, 95%CI 4.9–22.3). In the subgroup which was BCG vaccinated and foreign-born, 2.3 TST+/QFT− results were expected. Therefore 95.7% (51.7 out of 54) of TST+/QFT− results in this subgroup are explained by BCG vaccination and being foreign-born. If BCG vaccination and being foreign-born did not influence the probability of TST+/QFT− discordance, in the whole study group 18.6 of TST+/QFT− results would have been expected compared to 125 that were observed. This makes it likely that 85.1% (106.4 out of 125) of the TST+/QFT− observations do not indicate LTBI. If BCG vaccination is considered separately, 85.5 (89.8 out of 105) of all TST+/QFT− results are explained by BCG vaccination.

**Table 4:** BCG vaccination or foreign born as putative cause for TST+/QFT− Discordance.

	TST+/QFT−		p-value	Odds Ratio*	95%CI
	Yes	No			
BCG	O (%) [E]	N (%)			
No	20 (3.4) [20]	565 (96.6)	<0.0005	1	–
Yes	105 (23.4)[15.2]	343 (76.6)		8.6	5.3–14.2
Born					
Germany	59 (7.7) [59]	712 (92.3)	<0,0005	1	–
foreign born	66 (25.2) [20.2]	196 (74.8)		4.1	2.8–5.8
BCG vaccination and born					
no BCG, born in Germany	8 (1.8) [8]	442 (98.2)	<0.0005	1	–
no BCG, foreign born	12 (8.9) [2.4]	123 (91.1)		5.4	2.2–13.5
BCG, born in Germany	51 (15.9) [5.8]	270 (84.1)		10.4	4.9–22.3
BCG, foreign born	54 (42.5) [2.3]	73 (57.5)		40.9	18.7–89.4
Total	125 (12.1)[18.6]	908 (87.9)			

* Gender, age, previous TST, and source population (HCW or contact tracing in general population) were not associated with TST+/QFT− discordance; TST+ is defined as TST > = 10 mm.

O = observed, E = expected.

TST−/QFT+ discordance was observed in 34 (3.3%) participants (table 2). Out of 100 cases positive in the QFT, 34 (34.0%) were negative in the TST. The probability of these discordant results increased with age (test for trend p<0.0005; table 5). It was not influenced by gender, working in the healthcare system, BCG vaccination, being foreign-born, and previous TST. For participants aged between 50 and 68 years, the odds ratio for a TST−/QFT+ discordance was 5.0 (95%CI 1.9–13.2) compared to those younger than 40 years. In young people a positive QFT which is not confirmed by TST is rare (1.7%, table 5). If the probability of TST−/QFT+ discordance was not influenced by age, 17.3 of these results would have been expected compared to the 34 observed. This makes it likely that 49.1% (16.7 out of 34) of the TST−/QFT+ observations can be explained by waning sensitivity of the TST with increasing age.

**Table 5:** Age as putative cause for TST−/QFT+ Discordance.

	TST−/QFT+		p-value	Odds Ratio*	95%CI
	yes	no			
Age	O (%) [E]	N (%)			
1–39 years	12 (1.7) [12]	704 (98.3)	<0.0005	1	-
40–49 years	14 (5.7) [4.2]	232 (94.3)		3.1	1.3–6.8
50–68 years	8 (11.3) [1.2]	63 (88.7)		5.0	1.9–13.2
Total	34 (3.3) [17.3]	999 (96.7)			

Gender, being foreign born, previous TST, BCG vaccination and source population were not associated with TST−/QFT+ discordance; TST+ is defined as TST ≥10 mm.

O = observed, E = expected.

35.9 (3.5%) discordant results (18.6 TST+/QFT− and 17.3 TST−/QFT+) remain unexplained by risk factors which yields a corrected agreement for the two test of 96.5%. This agreement corresponds with the agreement of the two tests in young people (under 40 years), born in Germany and not BCG vaccinated (table 6).

**Table 6:** Results of the QFT and the TST with a diameter of 10 mm or more in German born persons younger than 40 years who were not BCG vaccinated.

	TST > = 10 mm		All
	positive	negative	
QFT	N (%)	N (%)	N (%)
Positive	7 (3.9)	5 (2.8)	12 (6.6)
Negative	3 (1.7)	166 (91.7)	169 (93.4)
Total	10 (5.5)	171 (94.5)	181 (100.0)

TST >5 mm: Agreement 90.0%, Kappa 0.42, p<0.0005.

TST > = 10 mm: Agreement 95.6%, Kappa 0.61, p<0.0005.

TST > = 15 mm: Agreement 94.5%, Kappa 0.27, p<0.0005.

## Discussion

With our data we were able to analyze migration, BCG vaccination and age as risk factors for 159 discordant results in contacts of TB cases tested with TST and QFT simultaneously. The proportion of discordant results we observed in our pooled analysis was somewhat lower (15% instead of 24%) than that described in a recent meta-analysis [13]. This might be explained by a higher proportion of risk factors for discordance in the studies that gave rise to this meta-analysis, i.e. the proportion of participants with BCG vaccination was 59% in the meta-analysis and 43% in our pooled population. As in this meta-analysis, the combination of TST+/QFT− results dominated the discordant results. Most of these discordant results can be explained by BCG vaccination or birth in a foreign country, which might be an indicator for NTM infection [25]. Being foreign-born and BCG-vaccinated explained 95.7% TST+/QFT− results that occurred in this subgroup. This might be explained by repeated BCG-vaccination in juveniles or by older age at which vaccination is performed. Both increase the probability of a positive TST [27]. In Germany BCG vaccination was performed in newborns only while in other countries (e.g. Poland, Czech Republic, Slovakia, Turkey) BCG vaccination is repeated [23], [26]. In a meta-analysis [27] it was estimated that depending on the time spent between vaccination and testing (≤10 years or >10 years) 21% to 41% of those with a BCG vaccination after the first birthday had a positive TST (diameter 10+ mm) explained by BCG. These estimates were based on comparisons of the TST in unvaccinated and vaccinated populations. Based on our comparison of the TST with the QFT and using the same approach, 85.5% of all TST+/QFT− results in vaccinated participants are most likely attributable to the BCG vaccination.

As a limitation of our study, we had no information on the age at vaccination, revaccination or exposure to NTM. Therefore we were not able to analyze the influence of these factors on TST. But we believe it to be likely that the observed interaction between being foreign-born and BCG vaccination might be explained by these factors.

Birthplace was a risk factor for a positive TST and QFT as well as for TST+/QFT− discordance in our data. Similar results were observed in US Navy recruits [14]. For the TST no positive correlation with age is seen in our data. Therefore it is unlikely that these TST+/QFT− results are explained by old infections due to the higher exposure in the countries where the immigrants were born (Turkey, East-Europe, Africa). In the Navy study it was shown that NTM infections (*M. avium*) were 5 times more likely in recruits born outside the US [14]. The effect of BCG vaccination could not be analyzed in the Navy study because none of the US-born recruits were vaccinated and therefore vaccination and being born in a country with high TB incidence was strongly correlated. Our data allow for combining birthplace and BCG vaccination. This allows us to analyze the effects of migration and BCG vaccination independently and to analyze the combined effect of migration and BCG vaccination. BCG vaccination might be associated with TB incidence in the sense that countries with high incidence continue vaccination or revaccination [23]. Therefore TST+/QFT− results might be explained by resolved or old TB infections that are detected by TST and not by QFT [28]. This hypothesis is not supported by our data. BCG vaccination is a strong predictor for TST+/QFT− results not only in foreign-born but also in German-born participants and the assumed association between age and resolved or old LTBI is found with the QFT but not with the TST.

Age is a strong predictor for a positive QFT that is not confirmed by the TST. In young people TST−/QFT+ results are rare. In 856 Navy recruits (mean age 20 years) no TST−/QFT+ combination was found [14]. We observed 34 of these combinations that mainly occurred in older participants. Because age is also a predictor for a LTBI it is likely that these discordant results are due to a higher waning of the T-cell mediated immune response to TST than to QFT. Our observation is indirectly confirmed by a Japanese study in which the association between age and LTBI was shown for IGRA but not for TST [29]. So far the immunologic interpretation of this observation is not clear. Either the QFT is positive because it is more sensitive to an old TB infection or the skin loses its capability to react and therefore both former and recent infections do not result in a positive TST with the same likelihood than in younger persons. Comparison of TST sensitivity in patients with active tuberculosis showed that TST sensitivity gets weaker with increasing age of the patient [30]. Thus it is likely that with increasing age the TST not only does not react to former infections but also is less sensitive to recent infections. This might either be due to difficulties to apply the tuberculin correctly into the aging skin or by decreasing mobility of the T lymphocytes that have to migrate to the forearm were the test is applied. The waning of the specific interferon-gamma response after years of tuberculosis infection was described in a Japanese population based on estimates of the expected prevalence of LTBI [18]. Our data suggest that waning is higher with the TST than with the QFT. The hypothesis that TST might be more sensitive to old infections while the IGRA mainly indicates recent infections is not supported by our data.

So far two prospective studies investigating the progression to active TB have been published [15], [31]. In the German study [15], progression to active TB was observed in those with a positive QFT only while in the Gambian study active TB was observed during the follow-up in 2 contacts, negative in the ELISPOT but positive in the TST at baseline [31]. Therefore so far the risk of progression to active TB can not be ruled out in TST+/QFT− contacts. However, based on our data, it is ikely that the proportion of those with a TST+/QFT− result that are at potential risk to progress towards active TB is rather small.

The proportion of discordant results that can not be explained by BCG vaccination, being foreign-born or by age is rather small (4.4%), indicating little potential for false-negative or false-positive QFT results. These findings support the hypothesis that the QFT is the test of choice in populations with a high BCG vaccination rate or with an increased chance of exposure to non-tuberculous mycobacteria (NTM), or in people older than 40 years. In young persons not vaccinated and unlikely to be exposed to NTM, the TST and the QFT are of comparable quality and the agreement between the two tests is above 95%. In older populations the TST is less sensitive than the QFT and in the German population with a migration background and/or a BCG vaccination the TST is far less specific than the QFT. In Germany, HCW with regular contact to TB patients are surveyed and in the general population contacts to TB patients are traced [32]. Using IGRA instead of TST would save HCW or other TB contacts from unnecessary follow-up [33]. Following our data, about 40% of migrants with BCG vaccination would profit from replacing TST by IGRA.

In conclusion, according to our data, it is not likely that the TST is more sensitive to old LTBI than the IGRA. Therefore we would like to suggest the use of an IGRA as the first test after exposure to a patient with active TB and in periodic screening for LTBI among exposed HCW, especially for those of foreign birth.
